# Deep-ultraviolet nonlinear optical crystals: concept development and materials discovery

**DOI:** 10.1038/s41377-022-00899-1

**Published:** 2022-07-01

**Authors:** Lei Kang, Zheshuai Lin

**Affiliations:** Functional Crystals Lab, Technical Institute of Physics and Chemistry, Chinese Academy of Sciences, Beijing, 100190 China

**Keywords:** Nonlinear optics, Optical materials and structures

## Abstract

Deep-ultraviolet (DUV, wavelength *λ* < 200 nm) nonlinear optical (NLO) crystal is the core component of frequency conversion to generate DUV laser, which plays an important role in cutting-edge laser technology and fundamental science. Significant progress has been made in both experimental exploration and theoretical design in the field of DUV NLO crystals over the past three decades. In-depth insight into “structure-property correlations”, in particular, allows for rigorous and precise identification of DUV NLO crystals. In this article, we reviewed the current experimental and theoretical research progress while elucidating the core concepts and stringent criteria of qualified DUV phase-matched second-harmonic generation crystals. We also discussed the development of the DUV NLO “structure-property correlations” from first principles and how it has sparked interest in related materials, as well as future directions for obtaining potential DUV NLO crystals.

## Abstract (web summary)

Structure-property correlations and related materials discovery of deep-ultraviolet nonlinear optical crystals are reviewed while elucidating the core concepts and stringent criteria.

## Graphical abstract

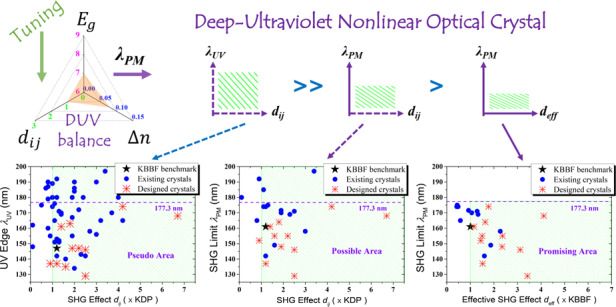

## Introduction

Nonlinear optics is closely related to laser technology since nonlinear optical (NLO) effects are usually evident in intense light. Shortly after Maiman demonstrated the first working laser in 1960^1^, Franken et al. carried out the first laser-driven NLO experiment in 1961^2^, in which the second harmonic generation (SHG, with the wavelength *λ*_2_ = 347 nm) of light by a ruby laser pulse (with the fundamental wavelength *λ*_1_ = 694 nm) in a quartz crystal was observed. After >60 years of development, nonlinear optics has penetrated various fields of modern optics and laser technology, which plays an increasingly important role in many scientific and high-tech fields such as all-solid-state lasers, ultrafast lasers, spectrometers, optical storage, and computing, by means of frequency conversion, electro-optic modulation, photorefractive effect, etc^3–9^. Up to now, the NLO technology is the most mature method to shift or extend the limited wavelength range that practical laser sources can directly access.

Practical NLO technologies depend heavily on the availability of NLO crystals^10^. Especially in the deep-ultraviolet (DUV, *λ*_2_ < 200 nm) spectral region, NLO crystals are the core components for generating stable and high-power DUV lasers, which have important applications in cutting-edge technologies such as medical, micromachining, lithography, photochemistry, spectroscopy, and microscopy^11–19^. When using DUV NLO crystals, SHG is the preferred method since it is technically efficient and convenient to produce DUV coherent light^20^, exhibiting more advantages in practical applications compared with other NLO processes, e.g., sum-frequency generation^10^. For a high-efficiency SHG output, the NLO crystals are required to meet strict criteria^21–23^. According to the SHG formula, under non-depleted pump approximation, the SHG intensity *I*_2_ can be written as^10^:

1 $$I_2\left( z \right) = \frac{{d_{eff}^2}}{{8\varepsilon _0cn_2n_1^2\lambda _2^2}}I_1^2z^2sinc^2\left( {\frac{{\Delta k \cdot z}}{2}} \right)$$

where *ε*_0_ is the vacuum dielectric constant, *c* is the light velocity in vacuum, *n*_1_ and *n*_2_ are the refractive indices at the fundamental and SHG frequencies ω and 2ω, respectively, and *I*_1_ is the intensity of fundamental light. Clearly, the SHG conversion efficiency *I*_2_/*I*_1_ is mainly determined by the transparent wavelength *λ*_2_ (DUV crystal requires the shortest *λ*_2_, i.e., UV absorption edge *λ*_UV_, <200 nm), the effective SHG coefficient *d*_eff_ (largely deduced from the SHG coefficients *d*_*ij*_), and the phase mismatch parameter ${{\Delta }}k = k_2 - 2k_1 = (n_2 - n_1)/\lambda _2$ (for convenience, consider type-I phase matching). Only in the case of Δ*k* = 0, i.e., under the phase-matching (PM) condition, does the SHG intensity increase quadratically with the light propagation distance *z* in the NLO crystal and have the largest conversion efficiency.

In case of SHG, the PM condition requires *n*(2ω) = *n*(ω)^9^. Except in very special cases, the optical refractive dispersion in materials does not allow to fulfil such a condition. To overcome this problem, a technique for phase-velocity matching at the conversion frequency of the fundamental and SHG waves was proposed, i.e., by exploiting the difference in the refractive indices of different polarization states of an optically anisotropic uniaxial or biaxial NLO crystal^24,25^. This technique highlighted one of the most crucial concepts in NLO crystals, i.e., birefringent phase matching, which is vital in the DUV region^21^. Afterwards, the birefringence Δ*n* is incorporated as a critical criterion into the essential evaluation of qualified NLO crystals, and a sufficiently large Δ*n* is required to achieve the PM condition (including propagating at a reasonable PM angle).

For quartz crystals, the SHG conversion efficiency of ruby laser at 694 nm is extremely low due to its small Δ*n* (<0.01), despite its *d*_36_ = 0.3 pm V^−1^. In a comparison, KH_2_PO_4_ (KDP) with larger Δ*n* (~0.04–0.05) can realize much stronger SHG intensity, which is often used as the benchmark for good NLO crystals (i.e., *d*_*ij*_ should be at least comparable to *d*_36_ = 0.39 pm V^−1^ of KDP)^26^. However, with the demand for DUV SHG, KDP cannot meet the requirement because its Δ*n* is still too small to satisfy the DUV PM condition. Shortwave transparent NLO crystals with superior PM capabilities are urgently needed, especially for practical 193.7 and 177.3 nm DUV lasers. Fulfilling this need, i.e., the discovery of DUV-transparent NLO crystals with sufficiently large Δ*n*, is scientifically significant and challenging.

From Eq. (1), it can be concluded that the crystal with good DUV NLO performance needs to have a wide energy bandgap *E*_*g*_ (at least > 6.3 eV to transmit DUV light) and a large SHG coefficient (*d*_*ij*_ > *d*_36_ of KDP) as well as a sufficient Δ*n* (preferably >0.06 default at 400 nm)^22^. The question then is which structural motifs in crystals can have wide *E*_*g*_, strong *d*_*ij*_, and large Δ*n* simultaneously, as *E*_*g*_ is in general inversely proportional to Δ*n* and *d*_*ij*_. Historically, the anionic group theory proposed by Chuangtian Chen has answered this question well, which tells us that the UV/DUV NLO properties of a crystal mainly depend on the composition and polarization arrangement of microscopic anionic groups and can be accurately evaluated by quantum chemical methods^27^. Guided by this theory, a set of concise and efficient structure-property relationships were summarized, and excellent predictability has been obtained especially in borate systems^27^. β-BaB_2_O_4_ (BBO) and LiB_3_O_5_ (LBO) are two important UV NLO crystals thus discovered^10^. BBO exhibits excellent UV NLO performance with wide *E*_*g*_ (~6.7 eV), strong *d*_*ij*_ (~4×KDP) and large Δ*n* (~0.10). However, due to the phase mismatch induced by insufficient refractive dispersion near the absorption edge (*λ*_UV_ ~185 nm), its shortest PM SHG output wavelength *λ*_PM_ ~205 nm > 200 nm so it cannot achieve available DUV SHG output^10^. For LBO, its *λ*_PM_ can only reach 277 nm due to the small Δ*n* (~0.04), which is also DUV-SHG unavailable^10^.

To further explore DUV NLO crystals, Chuangtian Chen et al. introduced beryllium into the borate structures^20^. Beryllium is really a magic element that can not only saturate the dangling bonds in boron-oxygen groups well to increase *E*_*g*_, but also rarely deteriorate the Δ*n* and *d*_*ij*_ of the layered borate groups, thus enabling balanced DUV NLO performance. It is no exaggeration to say that without beryllium there would be no discovery of KBe_2_BO_3_F_2_ (KBBF) and no development of DUV NLO crystals^27^.

KBBF is the first DUV SHG crystal that can break the “200-nm-wall” as its *λ*_PM_ ~161 nm^20^. In KBBF, all planar anionic (BO_3_)^3−^ groups are aligned in the same orientation to achieve a strong *d*_*ij*_, and their dangling bonds are saturated with Be^2+^ cations to enlarge *E*_*g*_. Meanwhile, the parallel-aligned (Be_2_BO_3_F_2_)_∞_ layers exhibit strong structural anisotropy for sufficiently large ∆*n*. Indeed, KBBF exhibits an excellent DUV NLO balance with *E*_*g*_ ~8.45 eV, *d*_22_ ~0.47 pm V^−1^, and Δ*n* ~0.088^10^. Furthermore, for the sixth-harmonic-generation 177.3-nm DUV lasers based on the practical 1064-nm pumping, KBBF exhibits a large *d*_eff_ ( = *d*_22_×cos*θ*_PM_×sin3*ψ*, where the PM angle *θ*_PM_ = 69°, and *ψ* can be tuned in the actual situation), which has promoted cutting-edge progress in Raman and angle-resolved photoemission spectroscopies for the measurements of super-fine structures, superconductivity, topological surface states, etc^12–19^. However, the layered KBBF exhibits heavy growth difficulties of large-sized single crystal due to weak interlayer interactions, requiring the use of prism-coupling technique to ensure its wide application range^20^. The lack of thick single crystals of KBBF with high optical quality has always hindered practical applications. Given the demand for DUV NLO crystals, it is imperative to search for new crystals comparable to or even beyond KBBF, and relevant research has been advancing^28–31^.

In the following, we first elucidate the conceptual basis of DUV NLO crystals with emphasis on current experimental and theoretical advances (Section II). We then discuss in detail how the “structure-property correlations” developed from first-principles calculations facilitate the structural exploration of novel DUV NLO crystals (Section III). Finally, we prospect the bottleneck issues and foresee the future development directions of practical DUV NLO crystals (Section IV).

## Development of conceptual basis for DUV NLO crystals

In the Preface of his classic book *Nonlinear Optical Borate Crystals*^10^, by reviewing the establishment of the anionic group theory and the development of NLO crystals, Chuangtian Chen pointed out that “the experience gained in this period benefited me a great deal because it helped me understand that becoming a useful NLO crystal depends not only on NLO coefficient of the crystal but also on its linear optical properties, such as birefringence, absorption edge, optical homogeneity, and damage threshold, as well as the physical-chemical properties of the crystal. Unfortunately, some physicists always tend to pay attention to χ^(2)^ only and seem to ignore other important parameters when searching for new NLO materials”. While the NLO effect is a prerequisite, it is linear optical properties, including *E*_*g*_, refractive indices *n*_*i*_, and Δ*n*, etc., that ultimately determine the actual performance of a DUV NLO crystal. It can also be explicitly manifested from Eq. (1).

**Fig. 1:**
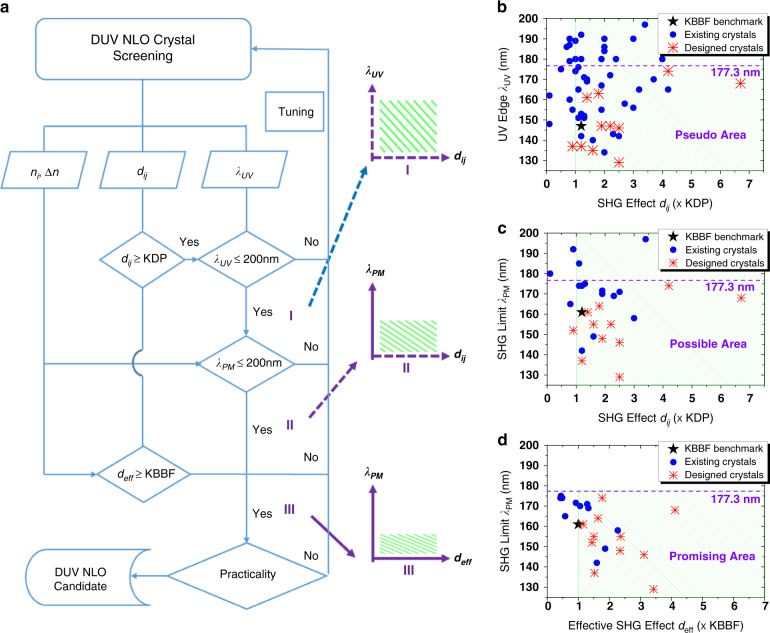
Key performance criteria of DUV NLO crystals and related materials. Screening scheme of DUV NLO crystal candidates (**a**), key criteria coordinates (I, II, III), and corresponding structures of “pseudo” (**b**), “possible” (**c**), and “promising” (**d**) DUV NLO crystals

Since the discovery of KBBF in the 1990s, numerous compounds have been synthesized, ranging from borates to phosphates, carbonates, and nitrates, and their linear and NLO properties have been determined by experiments and/or first-principles calculations^27–57^. Following the core concepts, we illustrate a screening scheme for DUV NLO crystal candidates based on the key criteria presented in Eq. (1), as shown in Fig. 1a. According to the “onion-peeling” screen, by judging “*Yes*” or “*No*” to key criteria including *d*_*ij*_, *d*_eff_, *λ*_UV_, and *λ*_PM_, most of the reported compounds (see Table S1 in the Supplementary Information for details) can be catalogized into the material areas shown in Fig. 1d, corresponding to three different evaluation coordinates, i.e., I, II, and III, respectively. If the criteria of coordinate I are satisfied, the requirement of DUV (*λ*_UV_ < 200 nm) and NLO (*d*_*ij*_ > KDP) is ostensibly met. However, in practice, DUV SHG output may not be achieved due to insufficient PM capability. This area is tentatively referred to as the “pseudo” area (Fig. 1b). Further considering the birefringent phase matching, i.e., substituting *λ*_PM_ for *λ*_UV_, coordinate II corresponds to the “possible” DUV NLO material area (Fig. 1c). However, this area may not be sufficient for practical high-power performance, especially due to the small effective SHG output. Only meeting the large *d*_eff_ and short *λ*_PM_ at coordinate III can be a theoretical “promising” material area (Fig. 1d). This is also the goal of current exploration. Specific examples are given below in conjunction with Fig. 1 and Table 1.

**Table 1:** Linear and NLO properties of typical UV and DUV NLO crystals

Crystals		*E*_*g*_ (eV)	*d*_*ij*_ (pm V^−1^)	×KDP	Δ*n* @ 400 nm	*λ*_UV_ (nm)	*λ*_PM_ (nm)	*d*_eff_^a^ (×KBBF)
KH_2_PO_4_	Exp.	7.14	*d*_36_ = 0.39	1.0	0.045	174	262	n/a
	Cal.	7.05	*d*_36_ = 0.40	1.0	0.046	176	261	n/a
LiB_3_O_5_	Exp.	7.85	*d*_31_ = 0.98; *d*_32_ = 1.05	2.7	0.043	158	277	n/a
	Cal.	7.75	*d*_31_ = 0.67; *d*_32_ = −0.72	1.8	0.051	160	251	n/a
β-BaB_2_O_4_	Exp.	6.56	*d*_22_ = 1.60	4.1	0.125	189	205	n/a
	Cal.	6.49	*d*_22_ = 1.57	4.1	0.113	191	196	n/a
KB_5_O_12_H_8_	Exp.	7.72	*d*_33_ = 0.05	0.1	0.064	162	217	n/a
	Cal.	7.43	*d*_33_ = 0.08	0.2	0.061	167	207	n/a
KBe_2_BO_3_F_2_	Exp.	8.45	*d*_22_ = 0.47	1.2	0.088	147	161	1.00
	Cal.	8.31	*d*_22_ = 0.41	1.1	0.059	150	172	0.50
RbBe_2_BO_3_F_2_	Exp.	8.18	*d*_22_ = 0.45	1.2	0.078	152	174	0.48
	Cal.	8.21	*d*_22_ = 0.40	1.0	0.057	151	185	n/a
CsBe_2_BO_3_F_2_	Exp.	8.23	*d*_22_ = 0.50	1.3	0.064	151	202	n/a
	Cal.	8.23	*d*_22_ = 0.38	1.0	0.055	151	210	n/a
NH_4_Be_2_BO_3_F_2_	Exp.	8.12	~1.2×KDP	1.2	0.091	153	174	0.46
	Cal.	8.37	*d*_22_ = 0.40	1.0	0.053	148	188	n/a
γ-Be_2_BO_3_F	Exp.	≥6.30	~2.3×KDP	2.3	–	≤200	–	–
	Cal.	8.88	*d*_12_ = 0.63	1.6	0.094	140	149	1.87
NH_4_B_4_O_6_F	Exp.	7.97	~3×KDP	3.0	0.130	156	158	2.27
	Cal.	7.87	*d*_32_ = 0.72; *d*_33_ = −0.91	1.8	0.112	158	164	1.40
KCaCO_3_F	Exp.	≥6.30	~3.3×KDP	3.3	–	≤200	–	–
	Cal.	6.31	*d*_11_ = 1.33	3.4	0.112	197	197	n/a
CaB_8_O_15_H_4_	Exp.	≥6.30	~1.4×KDP	1.4	–	≤200	–	–
	Cal.	7.43	*d*_23_ = 0.41	1.1	0.093	165	174	0.42
Be_2_BO_5_H_3_	Cal.	8.20	*d*_22_ = 0.52	1.3	0.087	152	175	0.44
PNF_2_	Cal.	8.80	*d*_32_ = 0.44; *d*_33_ = 0.86	1.2	0.164	142	142	1.60
Be_2_CO_3_F_2_^b)^	Cal.	8.43	*d*_22_ = 0.87	2.2	0.111	147	155	2.37
KAlCO_3_F_2_^b)^	Cal.	8.43	*d*_22_ = 0.73	1.9	0.108	147	148	2.35
PB_3_O_6_F_2_^b)^	Cal.	9.05	*d*_16_ = 0.45	1.2	0.099	137	137	1.52
BeB_2_O_4_^b)^	Cal.	9.05	*d*_36_ = 0.34	0.9	0.133	137	152	1.44

^a^For type-I phase matching at 177.3 nm. ^b^The structures are theoretically designed; “n/a” means *d*_eff_ is not applicable; “---” represents there are no available data

The first key criterion is the combination of *d*_*ij*_ (or those from powder SHG signals) and *λ*_UV_ as coordinated in Fig. 1b, which can be easily determined by experiments. However, this screening criterion is too coarse. Numerous materials with relatively large NLO effects (*d*_*ij*_ > KDP) appear to be DUV transparent (*λ*_UV_ < 200 nm), but they do not achieve effective PM output in the DUV region. Such materials are DUV-trivial and are essentially “pseudo” DUV NLO crystals. As marked in Fig. 1b, they are diverse and huge in number, not rare at all, but fail to show potential application value in actual DUV laser technology. This is mainly because the DUV transmission alone is not harsh, which can be easily met in conventional wide-*E*_*g*_ oxide materials, with similar examples abound.

The second key criterion encompasses the core concept of birefringent phase matching, which is currently the most critical technical requirement to achieve DUV PM output, although quasi-phase-matching could achieve DUV conversion through periodic ferroelectric polarization inversion as well. In earlier explorations, the Δ*n* greater than 0.06 was considered a suitable condition for phase matching in the DUV region^21,22^. But in some cases, e.g., CsBe_2_BO_3_F_2_ (CBBF)^58^, it fails due to inappropriate refractive dispersion^59^. Currently, the so-called DUV NLO crystal is at least a DUV NLO crystal with *λ*_PM_ < 200 nm and *d*_*ij*_ > KDP as shown in Fig. 1c. Even if the criterion seems to be satisfied, the actual DUV coherent conversion capability may still be insufficient, due to the small *d*_eff_ at particularly important DUV wavelengths (e.g., 193.7 or 177.3 nm). Such crystals are just “possible” DUV NLO crystals that appear to achieve DUV NLO performance, but still lack advantages in practice and are not “promising” DUV NLO crystals. For example, RbBe_2_BO_3_F_2_ (RBBF) has comparable *d*_22_ ~0.5 pm V^−1^ as KBBF and exhibits short *λ*_PM_ ~174 nm, but its *d*_eff_ at 177.3 nm is only half that of KBBF^60,61^, so its DUV conversion efficiency is insufficient considering the limited size of its crystal growth. Nonetheless, the DUV NLO performance of RBBF has surpassed those of most materials.

As a preliminary screening, the criteria of *λ*_PM_ < 200 nm and *d*_*ij*_ > KDP have great reference value, which can be efficiently evaluated by combining calculations and experiments, and the scope of potential materials can be quickly locked. Accurate localization based on this screening requires stricter theoretical and practical criteria for further growth preparation and evaluation of large-sized crystals. In particular, a material with large *d*_*ij*_ does not necessarily has a large *d*_eff_, as the core effect shown in Eq. (1), which is not only determined by the SHG coefficient *d*_*ij*_ but also largely depends on the PM wavelength *λ*_PM_ and the PM angle *θ*_PM_^10^. Under current technical conditions, there are only materials that can achieve effective DUV PM output (e.g., *λ*_PM_ < KBBF) with sufficient NLO conversion efficiency (e.g., *d*_eff_ > KBBF), as shown in Fig. 1d, especially for 193.7 and 177.3 nm lasers, can be referred to as “promising” DUV PM SHG crystals.

In the experimental progress, γ-Be_2_BO_3_F (BBF) and NH_4_B_4_O_6_F (ABF) are two rare structures with theoretically “promising” NLO performance, as marked in the preferred area of Fig. 1d^43,50^. BBF exhibits a theoretically larger *E*_*g*_ (cal. ~8.88 eV), SHG effect (cal. *d*_12_ ~1.6×KDP) and Δ*n* (cal. ~0.09) than KBBF, so that its shortest PM SHG output *λ*_PM_ (cal.) can reach 149 nm^50^. Its *d*_eff_ at 177.3 nm is greater than KBBF, which meets the theoretical standard of DUV NLO crystals. ABF has a superior SHG effect (~3×KDP), Δ*n* (~0.1) and *λ*_PM_ (~158 nm) than KBBF^43^. Its effective SHG coefficient *d*_eff_ is twice that of KBBF, despite its *E*_*g*_ (~8 eV) is smaller than KBBF.

Note that the above discussion in Fig. 1 refers only to theoretical criteria, typically used to theoretically pinpoint the DUV NLO performance of a crystal. Pratical applications need to consider more technical factors, such as device properties of crystal growth, physical-chemical stability, mechanical-thermal functionality, machining, defect absorption, and laser damage, etc^10^. Only by considering these theoretical and practical criteria can we finally assess whether the material is a pratical DUV NLO crystal. It is precisely because of satisfying the comprehensive properties that KBBF becomes an excellent DUV NLO crystal.

## First-principles exploration of new DUV NLO crystals

To this day, KBBF, as the first DUV SHG crystal discovered, remains the best DUV NLO crystal. It can achieve the shortest PM SHG output of 161 nm and has achieved the 177.3-nm DUV laser of 200 mW^62,63^. However, the development of DUV laser technology requires DUV NLO crystals with shorter SHG output wavelengths and higher SHG conversion efficiency to meet the needs of high-performance lasers with higher precision and higher power^64^. Therefore, it is necessary to continuously explore new DUV NLO crystals to achieve this goal.

Unfortunately, prior to 2013, no materials were discovered that might exceed the DUV NLO properties of KBBF, such as achieving the *λ*_PM_ shorter than 161 nm^65^. Based on years of extensive materials exploration, among the structural systems known at the time, the KBBF structure has pushed DUV NLO performance to the theoretical limit^65^. As such, it is a great scientific challenge to continue to improve DUV NLO performance beyond KBBF. To address this challenge, over the past decade several design strategies have been proposed to promote the performance improvement of DUV NLO crystals^66–69^. By combining advanced first-principles modeling and simulations, a series of potential DUV PM SHG crystals were evaluated, designed, and predicted^69–75^, some of which have been partially verified experimentally^50,76^.

The adopted first-principles approach has practical predictivity for the micro-structures and macro-properties of functional crystalline materials, due to its superiority in the following aspects: (1) it can give the microscopic and intrinsic atomic and electronic characteristics of materials, so as to give a unified description of the properties of materials at a deeper level; (2) it can quickly obtain a large amount of information about material properties, and can effectively simulate extreme environments that are difficult to reach experimentally; and (3) it can design materials and simulate their properties according to the intention of the researchers and under the guidance of certain physical-chemical principles, to provide ideas and references for the experimental search.

Especially in the evaluation of DUV NLO crystals, the first-principles method has shown many advantages and has become an efficient, reliable, and cost-effective material-exploration technique^77,78^, which can quickly lock the range of potential materials for experimental exploration. Specifically, (i) the first-principles density functional theory (DFT) calculations based on advanced hybrid functional can obtain accurate optical *E*_*g*_, while in experiments large-sized bulk crystals are required to measure optical spectra below 190 nm. Meanwhile, (ii) the DFT calculation with scissors correction can obtain accurate linear (*n*_*i*_ or Δ*n*) and NLO (*d*_*ij*_) properties and can strictly evaluate the PM capability and effective SHG output^77^. Experimentally, large-sized crystals are required to characterize the refractive indices and SHG coefficients in different polarized directions, although the powder SHG effect can roughly screen the SHG intensity^26^. Furthermore, (iii) the first-principles simulations can elucidate the micro-structural SHG origin^79^, understand the mechanism of NLO generation, and then regulate or improve the NLO performance, enabling theoretically guided DUV NLO material design^78^.

Based on the proposed first-principles approach (see Computational Methods in the Supplementary Information for details), we have calculated the linear and NLO properties of classical NLO phosphates and borates, e.g., KDP, BBO, LBO, exhibiting high computational accuracy as listed in Table 1. On this basis, we further studied the KBBF-family materials, including KBBF, RBBF, and CBBF, and successfully explained the intrinsic reason why CBBF cannot achieve the DUV SHG output depending on the “NLO size-effect”^66^.

### Tuning of the interlayer cations

KBBF, RBBF, and CBBF have similar structures with comparable *λ*_UV_ ~150 nm and *d*_22_ ~1.2×KDP. However, their PM SHG capabilities in the DUV region are quite different due to different Δ*n*. As shown in Table 1, the Δ*n* of RBBF is 0.01 smaller than that of KBBF. It is this slight difference that makes the *λ*_PM_ of RBBF (~174 nm) red-shifted by 13 nm compared to KBBF (~161 nm). As for CBBF, although it is transparent up to 151 nm, its Δ*n* is 3/4 of that of KBBF, so that its *λ*_PM_ is only 202 nm, which can no longer achieve effective DUV SHG output^58^. In fact, the KBBF-family already contains the connotation of DUV NLO crystals as shown in Fig. 1, i.e., KBBF stands for a qualified and “promising” DUV NLO crystal, RBBF for “possible” and CBBF for “pseudo”. The fundamental core of these differential classifications is the birefringent phase matching and its induced *d*_eff_. Therefore, it is necessary to first explain the birefringent origin and difference.

**Fig. 2:**
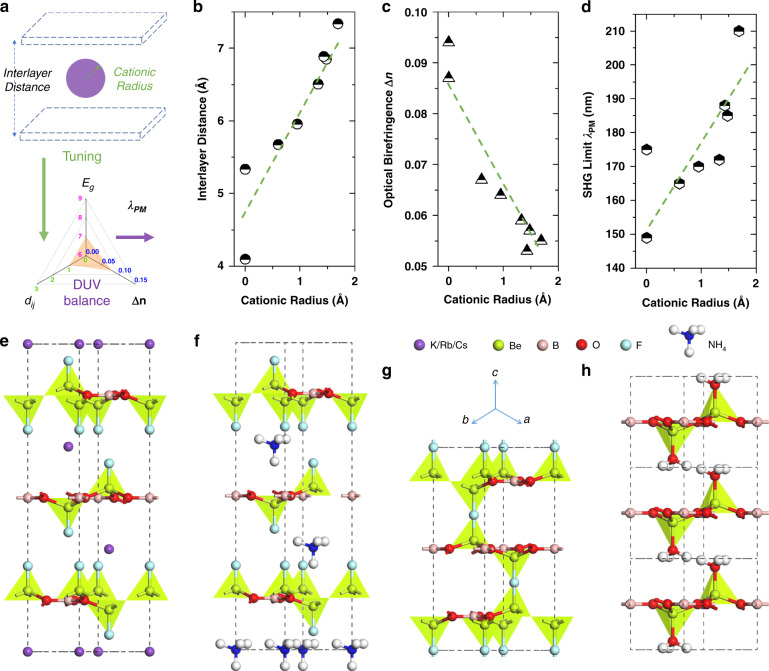
Interlayer cationic tuning strategy and related materials. Schematic diagram of tuning the DUV balance with A-site cationic size (**a**), and the theoretical data of effective interlayer distance (**b**), Δ*n* (**c**), and *λ*_PM_ (**d**) varying from the A-site cationic radius in the KBBF-type structures; structural evolution from KBBF with K^+^ (**e**) to NH_4_Be_2_BO_3_F_2_ (ABBF) with NH_4_^+^ (**f**), F-bridged γ-Be_2_BO_3_F (BBF) (**g**) and vdW Be_2_BO_5_H_3_ (BBH) without the A-site cations (**h**)

The Δ*n* originates from the difference between the intralayer and interlayer optically polarized susceptibility. In KBBF, the intralayer polarization is greater than the interlayer polarization, so the refractive index of ordinary light *n*_o_ is greater than that of extraordinary light *n*_e_, demonstrating that it is a negative uniaxial crystal. As the A-site cation changes from K^+^ to Rb^+^ and Cs^+^, the cationic radius between (Be_2_BO_3_F_2_)_∞_ layers increases, resulting in a decrease in the interlayer spacing and enhanced interlayer polarization, while keeping the intralayer polarization almost unchanged. As a result, the Δ*n* become smaller and the corresponding PM ability decreases. This is the so-called “NLO size-effect” induced by the A-site cationic size in the KBBF-type structures, as illustrated in Fig. 2a, d^66,68^.

Accordingly, rational tuning of the A-site cationic size has become an effective strategy to improve the DUV NLO performance, especially in the KBBF-type structures. Although the A-site cations themselves do not contribute much to the SHG and Δ*n*, they can affect the arrangement and density of the anionic groups, thereby tuning the overall Δ*n*, which in turn affects the final DUV NLO performance (Fig. 2a). A natural idea for improvement is to replace K^+^ with Na^+^ or Li^+^ with a smaller cationic radius^68^. The first-principles calculations demonstrate that there is indeed an improvement as plotted in Fig. 2b, d. However, no isomorphic NaBe_2_BO_3_F_2_ or LiBe_2_BO_3_F_2_ structures were experimentally obtained apart from a monoclinic NaBe_2_BO_3_F_2_ phase. Unfortunately, its performance is not as good as that of KBBF due to the irregular arrangement and the single-crystal growth ability is not improved^80^. If a hybrid cationic group could be introduced, thereby introducing hydrogen-bonds, it might improve the crystal growth ability while maintaining the DUV NLO performance. NH_4_Be_2_BO_3_F_2_ (ABBF) came into being, as shown in Fig. 2e, f, using NH_4_^+^ instead of K^+^^71^. The theoretical and experimental results show that it exhibits certain DUV NLO and crystal growth properties^50^. However, it is not better than KBBF, only reaching the performance level of RBBF (Table 1). Without larger crystal size than RBBF, the usefulness of ABBF is still limited.

Based on the NLO size-effect, it is conceivable that if all the potential of the cations can be tapped, the DUV NLO performance of a crystal may be further improved, thus breaking the KBBF limit. An extreme design strategy is to reduce the size of the A-site cations to zero, i.e., to eliminate the A-site cations^68^, thereby maximizing the DUV NLO potential. Such theoretical design was first validated in the F-bridge-connected γ-Be_2_BO_3_F (BBF) layered framework system as shown in Fig. 2g^50,71^. The first-principles results demonstrate that the shorter SHG output and the stronger SHG effect are indeed achieved (Table 1). However, Be_2_BO_3_F exists in three structural phases and is difficult to grow; the bulk single crystals of BBF have not been reported yet^50^.

Furthermore, another design strategy to eliminate the A-site cations, i.e., via van der Waals (vdW) connection, has been proposed, and a series of possible DUV NLO structures have been accordingly predicted^68,73,75,78,81^. Among them, an existing berborite (Be_2_BO_5_H_3_, BBH) mineral as shown in Fig. 2h can theoretically achieve DUV NLO performance comparable to KBBF^73^. The vdW DUV NLO crystal, firstly proposed by our group, holds great potential to extend the DUV NLO applications with higher output power and shorter output wavelength. The first-principles structural designs such as PB_3_O_6_F_2_ and SiCO_3_F_2_ are expected to exceed 150 nm and realize superior DUV NLO performance beyond KBBF in the application of ^229^Th nuclear clock^78,82^. However, their materials growth and preparation remain great challenges.

### Extending the anionic groups

In addition to tuning the A-site cations, the DUV NLO performance can also be improved by extending the anionic groups. Conventional DUV NLO materials are layered fluorine-based borate anionic frameworks, since the layered frameworks favors large Δ*n*, and the fluorine-based borate anionic groups favor large *E*_*g*_. To this end, the exploration of the material is extended from borate to carbonate anionic groups, from fluorine-based to hydroxyl-based anionic groups, and from layered to chained anionic groups, respectively^69,70,76^, to enhance or implement DUV NLO properties in multiple ways.

**Fig. 3:**
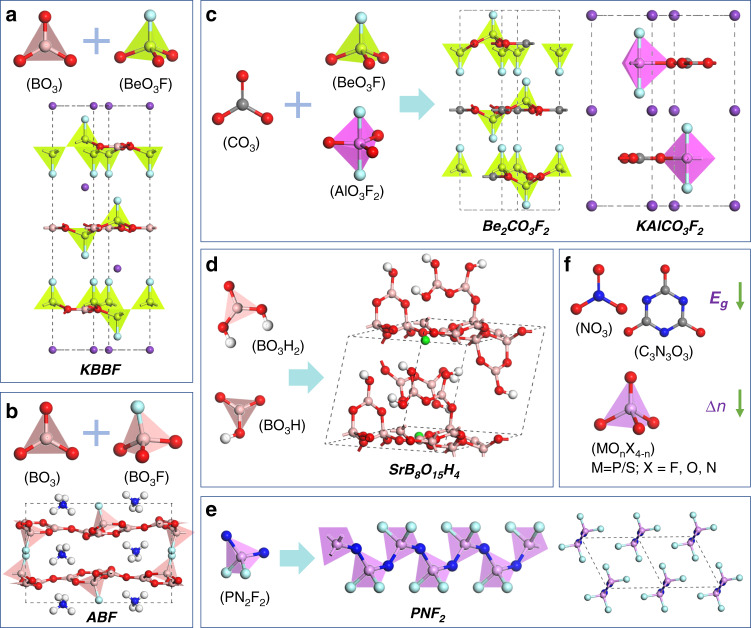
Anionic group extending strategy and related materials. Basic NLO motifs and structural expansion from KBBF (**a**) and NH_4_B_4_O_6_F (ABF) (**b**) to fluoride carbonates Be_2_CO_3_F_2_ and KAlCO_3_F_2_ (**c**), hydroxyborate SrB_8_O_15_H_4_ (**d**), poly(difluorophosphazene) PNF_2_ (**e**), and nitrates, cyanurates, phosphates, sulfates (**f**)

Anionic extension from borate to carbonate is a feasible scheme, as illustrated in Fig. 3a, c, since the planar triangular (CO_3_)^2−^ has large *d*_*ij*_ and Δ*n*, similar to (BO_3_)^3−^ with NLO-active π-orbitals^83^. However, most experimentally synthesized structures are alkali and alkaline earth metal carbonates (e.g., KCaCO_3_F) with insufficient DUV *E*_*g*_^83^. Through the first-principles analysis, we found that Be and Al can enlarge *E*_*g*_ by saturating the dangling bonds of (CO_3_)^2−^ while maintaining the balanced SHG effect and Δ*n*. Accordingly, a series of Be-based and Al-based carbonates were designed, showing potential DUV PM NLO performance^70^. Among them, KAlCO_3_F_2_ and vdW Be_2_CO_3_F_2_ as shown in Fig. 3c can exhibit shorter DUV SHG limits (~148 and 155 nm) and larger SHG effects (~0.73 and 0.87 pm V^−1^) than those of KBBF (~161 nm, 0.47 pm V^−1^) (Table 1)^78^.

The experimental findings of the ABF-series also depend on the theoretical study of the LiB_6_O_9_F system to a certain extent^84,85^, showing great potential of NLO fluorooxoborates^43,86^. As illustrated by Fig. 3b, d, the replacement of fluorine-based anionic groups with hydroxyl-based anionic groups can not only ensure large *E*_*g*_, but also favor hydrothermal or solution synthesis conditions suitable for crystal growth. For example, we discovered possible structures with DUV NLO properties in the hydroxyborate (e.g., SrB_8_O_15_H_4_) and hydroxycarbonate (e.g., LiZnCO_3_OH) systems by hydrothermal synthesis^55,76^. However, their performance is not comparable to KBBF (Table 1). Even replacing Zn with Be, the resulting LiBeCO_3_OH failed to surpass the KBBF performance. Nevertheless, these theory-parallel experimental studies at least demonstrate the feasibility of hydroxyborates and hydroxycarbonates in DUV NLO materials, especially if improved crystal growth properties are exhibited.

Anionic extension from layer to chain is another feasible scheme as illustrated in Fig. 3e, since chained anionic groups have lower dimensionality and greater structural anisotropy than layered anionic groups. Through the first-principles analysis, we investigated the isolated and polymer chain-like structures, which exhibit greater optical anisotropy and structural polarity, leading to greater Δ*n* and SHG effects^69^. If the *E*_*g*_ can be further increased, the DUV NLO performance can be achieved. Accordingly, the existing poly(difluorophosphazene) (PNF_2_) was predicted, showing larger *E*_*g*_ (~8.8 eV), stronger *d*_*ij*_ (~0.86 pm V^−1^), larger Δ*n* (~0.16) and shorter *λ*_PM_ (~142 nm) than KBBF (Table 1)^69^. It is the first predicted chain-like polymer DUV NLO structure.

The extending of other anionic groups has also led to the emergence of new materials such as nitrates, cyanurates, phosphates, and sulfates^57,87–90^. Except for a few special structures^78^, most of them usually fail to achieve the “possible” or “promising” DUV NLO performance balance, because of either reduced *E*_*g*_ or decreased Δ*n* as illustrated in Fig. 3f. In general, they are not yet candidates in the preferred area for DUV NLO materials exploration.

In short, the proposed first-principles approach can not only characterize the properties and mechanisms of DUV NLO crystals, but also elucidate the “structure-property correlation” laws, which provide insights for performance improvement and materials design^77^. Meanwhile, it must be recognized that the first-principles methods have applicability and cannot be used illegally. Although the calculations of linear and NLO properties are independent of experiments, the computational parameters need to be self-consistent and verified by existing experimental results. A class of parameters including energy cutoff, k-point mesh, and the number of conduction bands must be tested specifically, because different structures and components require different precisions to ensure convergence. In particular, the number of conduction bands is extremely important to keep performance convergent, otherwise, problems will arise (see Computational Methods in the Supplementary Information for details). This class of parameters is not adjustable in principle. The better the parameters, the higher the accuracy, so it is necessary to keep the balance between accuracy and efficiency as much as possible. Another class of parameters, such as functionals or pseudopotentials, depends on different compound systems. In particular, the description of *E*_*g*_ by the hybrid functionals must in principle be comparable to a similar system. The hybrid parameters need to be corrected with the experimental values, and the computational methods must be kept uniform. Only then can the calculations be comparable and describe qualitative trends. Failure to control variables creates potential errors. Recognizing the applicability of first-principles approaches to specific results on performance and structural stability, it is necessary to incorporate experimental feedback and optimization to ensure predictable trend descriptions. Overall, the first-principles calculations can provide an important reference for DUV NLO evaluations, but final performance determinations also require rigorous optical characterization of large-sized crystals rather than relying solely on theoretical results. Only by recognizing the theoretical applicability can the theoretical predictability be maximized.

### Outlook of DUV NLO crystals

In summary, DUV NLO crystals have strict concepts and systematic criteria; it is why they are so rare. Numerous NLO materials are just transparent in the DUV region, but they cannot achieve effective DUV PM output; they are essential “pseudo” DUV NLO crystals. A few crystals appear to meet the DUV NLO performance criteria, but their actual DUV coherent conversion capabilities are insufficient especially for SHG; they basically belong to “possible” DUV NLO crystals. Currently, only crystals capable of achieving effective DUV PM output with sufficient SHG conversion efficiency are called “promising” DUV NLO crystals. It should be emphasized that the materials exploration of DUV NLO crystals must rely on these strict concepts and self-consistent criteria. Without meeting the concepts, it is not strictly a DUV NLO crystal; without meeting the criteria, it cannot achieve a truly efficient DUV coherent output.

Moreover, high-quality large-sized single crystals are the fundamental and ultimate destination of NLO crystals. Reasonable performance evaluation is a crucial step for NLO crystals before large-sized crystal growth to avoid unnecessary time and financial consumption. The combination of theoretical analysis and experimental characterization is an effective way to explore NLO crystal materials. The anionic group theory-guided exploration of borate materials provides a successful example of this approach. With the development of high-performance computing, the first-principles-guided materials exploration and precise positioning have gradually entered the stage and have become more and more recognized. However, the practically important crystal growth and stability issues cannot be accurately analyzed from the first principles under current computational conditions. In comparison, it relies more on the development of experimental techniques and phenomenological theories.

With the deepening of research, the current exploration of DUV NLO crystals is faced with the following issues, which are also directions that need attention in the future: (1) it is difficult to grow carbonate crystals, and qualified large-sized crystals are rarely reported; (2) the *E*_*g*_ of nitrates or cyanurates are small for DUV SHG; (3) the Δ*n* of phosphates or sulfates are insufficient for DUV phase matching; (4) the biaxial or deliquescence difficulties in fluorooxoborates or hydroxyborates need to be overcome technically; (5) the harsh synthesis of composite anionic crystals such as borophosphates needs to be improved; (6) low-dimensional structures exhibit crystalline instability under practical conditions; etc. Currently, the beryllium-based borate structure is still an ideal candidate material system for DUV NLO crystals with comprehensive properties. Practical studies of other crystal systems may be a long way off. In addition, advances in quasi-phase matching or new phase matching continues^91,92^. The development of photonic crystals and metamaterials may bring new opportunities for the design of NLO crystals^93,94^. All in all, after more than ten years of development, although possible structures with DUV NLO properties or even exceeding KBBF have been discovered from time to time, there are basically no practical crystals and no effective DUV harmonic output being reported. All these advances just show the great challenges in the discovery of DUV NLO crystals. In other words, if the performance of KBBF cannot be broken, then carefully growing KBBF crystals or exploring new crystal growth techniques may be an unwieldy but effective route.

In conclusion, we have reviewed the application background and actual concept development of DUV NLO crystals. For better differentiation, we systematically analyzed the theoretical performance screening criteria and their practical implications for qualified DUV NLO crystals. A theoretically promising DUV NLO crystal with an effective DUV SHG output can only be achieved if stringent performance criteria are met. We further reviewed the driving role of the anionic group theory and first-principles methods in the design and prediction of DUV NLO crystals. In particular, the first-principles calculations have reflected the advancement and superiority in the research and improvement of “structure-property correlations”. These underlying correlations are useful and instructive for current and future DUV NLO materials exploration, especially when exploration faces bottlenecks. This review provides an important reference for the evaluation of DUV NLO performance, and would have a positive impact on conceptual clarification and materials exploration in the field of DUV NLO crystals.

## Supplementary information

Supplementary Information for Deep-ultraviolet Nonlinear Optical Crystals: Concept Development and Materials Discovery
