# Supplementary material for: Deep-ultraviolet nonlinear optical crystals: concept development and materials discovery
Source: Light Sci Appl. 2022 Jul 1;11:201. doi: 10.1038/s41377-022-00899-1 (PMC9249785; doi:10.1038/s41377-022-00899-1)
Supplement: Supplementary file 1 — Supplementary Information for Deep-ultraviolet Nonlinear Optical Crystals: Concept Development and Materials Discovery [file 41377_2022_899_MOESM1_ESM.docx]

*Supplementary Information for*

**Deep-ultraviolet Nonlinear Optical Crystals: Concept Development and Materials Discovery**

Lei Kang and Zheshuai Lin*

*Functional Crystals Lab, Technical Institute of Physics and Chemistry, Chinese Academy of Sciences, Beijing 100190, China*

*E-mail: zslin@mail.ipc.ac.cn

**Table S1**. Linear and nonlinear optical (NLO) properties including static second harmonic generation (SHG) coefficients *d_ij_*, effective SHG effects *d_eff_* at 177.3 nm for the type-I phase matching, ultraviolet (UV) absorption edges *λ_UV_*, and the shortest phase-matching (PM) SHG output wavelengths *λ_PM_* of typical existing and designed UV and deep-UV NLO crystals.

| Crystals | *d_ij_* (×KDP) | *λ_UV_* (nm) | *λ_PM_* (nm) | *d_eff_* (×KBBF) |
| --- | --- | --- | --- | --- |
| Existing Structures | | | | |
| KH_2_PO_4_ | 1.0 | 174 | 262 | n/a |
| BPO_4_ | 2.0 | 134 | >200 | n/a |
| Ba_3_ZnB_5_O_10_PO_4_ | 4.0 | 180 | 365 | n/a |
| KBe_2_BO_3_F_2_ | 1.2 | 147 | 161 | 1.00 |
| RbBe_2_BO_3_F_2_ | 1.2 | 152 | 174 | 0.48 |
| CsBe_2_BO_3_F_2_ | 1.3 | 151 | 202 | n/a |
| NaBe_2_BO_3_F_2_ | 1.1 | 151 | 185 | n/a |
| NH_4_Be_2_BO_3_F_2_ | 1.2 | 153 | 174 | 0.46 |
| α-Be_2_BO_3_F | 0.1 | 148 | 180 | n/a |
| γ-Be_2_BO_3_F | 1.6 | 140 | 149 | 1.87 |
| BaAlBO_3_F_2_ | 3.2 | 165 | 274 | n/a |
| Rb_3_Al_3_B_3_O_10_F | 1.0 | 189 | 235 | n/a |
| NaSr_3_Be_3_B_3_O_9_F_4_ | 2.2 | 172 | 248 | n/a |
| BaBe_2_BO_3_F_2_ | 0.1 | 148 | 180 | n/a |
| K_3_Ba_3_Li_2_Al_4_B_6_O_20_F | 1.5 | <190 | 243 | n/a |
| Li_2_B_6_O_9_F_2_ | 0.9 | 155 | 192 | n/a |
| NH_4_B_4_O_6_F | 3.0 | 156 | 158 | 2.27 |
| RbB_4_O_6_F | 0.8 | 160 | 165 | 0.58 |
| CsB_4_O_6_F | 1.9 | 155 | 171.6 | 0.92 |
| CsKB_8_O_12_F | 1.9 | 155 | 170 | 1.06 |
| Ca_2_B_10_O_14_F_6_ | 2.5 | 142 | 171 | 1.29 |
| Sr_2_B_10_O_14_F_6_ | 2.3 | 143 | 169 | 1.33 |
| KB_5_O_12_H_8_ | 0.1 | 162 | 217 | n/a |
| SrB_8_O_15_H_4_ | 1.1 | 176 | 185 | n/a |
| CaB_8_O_15_H_4_ | 1.1 | 165 | 174 | 0.42 |
| Be_2_BO_5_H_3_ | 1.3 | 152 | 175 | 0.44 |
| LiB_3_O_5_ | 2.7 | 158 | 277 | n/a |
| β-BaB_2_O_4_ | 4.1 | 189 | 205 | n/a |
| CsLiB_6_O_10_ | 2.4 | 180 | 238 | n/a |
| YAl_3_(BO_3_)_4_ | 3.7 | 170 | 220 | n/a |
| K_2_Al_2_B_3_O_7_ | 1.2 | 180 | 226 | n/a |
| Sr_2_Be_2_B_2_O_7_ | 4.2 | 165 | >200 | n/a |
| BaAl_2_B_2_O_7_ | 1.9 | 180 | >200 | n/a |
| β-KBe_2_B_3_O_7_ | 0.8 | 187 | 290 | n/a |
| γ-KBe_2_B_3_O_7_ | 0.7 | 186 | 243 | n/a |
| RbBe_2_B_3_O_7_ | 0.8 | 179 | 284 | n/a |
| Na_2_CsBe_6_B_5_O_15_ | 1.2 | 192 | 452 | n/a |
| Na_2_Be_4_B_4_O_11_ | 1.3 | 171 | 256 | n/a |
| LiNa_5_Be_12_B_12_O_33_ | 1.4 | 169 | 258 | n/a |
| CsB_3_O_5_ | 1.9 | 167 | >200 | n/a |
| Li_5_Rb_2_B_7_O_14_ | 0.8 | 190 | >200 | n/a |
| BaBPO_5_ | 1.0 | 180 | >200 | n/a |
| Li_3_Cs_2_B_5_O_10_ | 0.5 | 175 | >200 | n/a |
| Li_2_SrB_2_O_6_ | 2.0 | 186 | >200 | n/a |
| Li_2_B_4_O_7_ | 1.4 | 170 | >200 | n/a |
| Ba_3_B_6_O_11_F_2_ | 3.0 | 190 | >200 | n/a |
| Ca_5_(BO_3_)_3_F | 2.0 | 190 | >200 | n/a |
| K_3_B_6_O_10_Cl | 4.0 | 180 | >200 | n/a |
| KCaCO_3_F | 3.4 | 197 | 197 | n/a |
| YCO_3_OH | 2.0 | 184 | 300 | n/a |
| PNF_2_ | 1.2 | 142 | 142 | 1.60 |
| Designed Structures | | | | |
| KBeCO_3_F | 1.8 | 163 | 164 | 1.63 |
| KAlCO_3_F_2_ | 1.9 | 147 | 148 | 2.35 |
| AlNO_3_F_2_ | 4.2 | 174 | 174 | 1.77 |
| KSiB_3_O_6_F_2_ | 1.4 | 161 | 161 | 1.15 |
| PBO_3_F_2_ | 1.6 | 135 | 155 | 1.50 |
| PB_3_O_6_F_2_ | 1.2 | 137 | 137 | 1.52 |
| YCO_3_F | 6.7 | 168 | 168 | 4.12 |
| AlCO_3_F | 2.5 | 146 | 146 | 3.12 |
| Be_2_CO_3_F_2_ | 2.2 | 147 | 155 | 2.37 |
| SiCO_3_F_2_ | 2.5 | 129 | 129 | 3.42 |
| BeB_2_O_4_ | 0.9 | 137 | 152 | 1.44 |

“n/a” means *d_eff_* is not applicable.

**Computational Methods**

First-principles calculations can accurately predict the key properties of deep-UV (DUV) nonlinear optical (NLO) crystals without introducing experimental parameters, and can deeply elucidate their intrinsic origins and mechanisms, providing strong theoretical support for structural design and performance tuning.^1,2^ However, the accuracy of the first-principles approach is premised on its applicability, which requires rigorous testing of convergence and repeatability. In general, the self-consistent testing is directly related to the calculation software, approximation methods, pseudopotentials, energy cutoffs, *k*-point meshes, energy convergence thresholds, structural optimization schemes, number of empty bands, etc. The selection of them requires a uniform standard of comparability.

The present first-principles results are obtained using the plane wave pseudopotential method implemented in the CASTEP package (academic release version 7.02) based on the density functional theory (DFT).^3-5^ The local density approximation (LDA) and generalized gradient approximation (GGA) in standard DFT are usually employed to simulate the energies and orbitals of the electronic ground states.^6,7^ The Perdew–Burke–Ernzerhof (PBE) functional is preferred because it is more general and more consistent with experiments when dealing with van der Waals structural optimization.^8^ Advanced DFT functionals beyond LDA and GGA are also developed to accurately predict the energy bandgaps in solid state materials, typically including sX-LDA (screened exchange, plus LDA correlation) and hybrid HSE functionals (combination of PBE functional with a predefined amount of exact exchange).^9,10^ Based on our benchmark testing in borates, carbonates, phosphates, and other oxide systems, for PBE0, a special HSE functional, the bandgap predictions for DUV materials are more in line with experimental measurements.^11-13^ As a comparison, HSE06 or sX-LDA usually underestimates the bandgaps for DUV crystals (note that this was tested under CASTEP and varies by calculation software).

The optimized norm-conserving pseudopotentials are adopted for all constituent elements to describe the ion-electron interactions.^14^ The advantage of norm-conserving pseudopotentials is that they can be applied to almost all calculations, including HSE, phonon vibrations, thus guaranteeing the uniformity of all computational parameters. Different types of norm-conserving pseudopotentials are functionally similar but need to keep the choice of each element the same. The most common type of norm-conserving pseudopotentials of 00.recpot are usually used in our related calculations since they can apply to almost all elements.

For options of energy cutoff, Monkhorst-Pack *k*-point meshes and convergence thresholds, CASTEP does a good convergence test.^15^ For wide-gap (*E_g_* > 3 eV) semiconductors, the setting with fine precision can basically achieve good convergence. For elastic and phonon calculations, it is sometimes necessary to choose ultrafine or even higher precision. For example, for KBe_2_BO_3_F_2_, a kinetic energy cutoff of 1000 eV is chosen with Monkhorst-Pack *k*-point meshes spanning less than 0.04 per Å^3^ in the Brillouin zone. The cell parameters and atomic positions are further optimized using the quasi-Newton method. The convergence thresholds between optimization cycles for convergence of energy, force, stress, and displacement are set as 5.0×10^–6^ eV per atom, 0.01 eV Å^-1^, 0.02 GPa, and 5.0×10^–4^ Å, respectively.^16^

One of the most important parameters is the number of empty bands or conduction bands, the choice of which directly determines the accuracy of the optical properties.^17^ If the calculation results related to the number of empty bands fails to converge, they are not self-consistent in principle. Even if it agrees with the experimental value, it is neither repeatable nor predictable. For bulk materials, the common convergence requires that the number of empty bands is at least 3-5 times the number of valence bands.^17^ Certain structures require certain tests. For 2D materials, the choice of the number of empty bands is related to the vacuum thickness. For example, the number of empty bands in B_2_S_2_O_9_ is about 10 times the number of valence bands.^18^

Under the premise of the convergence of various calculation parameters, the refractive indices (*n* and the birefringence Δ*n*) and static SHG coefficients *d_ij_* can be obtained based on the scissors-corrected GGA-PBE method.^19-21^ Here the scissors operator is set to the difference between the PBE0 and GGA-PBE bandgaps, which is not an arbitrarily adjustable parameter but is strictly equal to the difference between the two bandgaps. This self-consistent *ab initio* approach has been shown to be an efficient method to study linear and NLO properties in a variety of NLO materials without introducing any experimental parameter. Especially for NLO materials with wide bandgaps, extensive test calculations on the linear and NLO properties suggest that this scissor-corrected PBE method can be used to obtain the accurate results in agreement with experiments.^1,2,22^ As such, the first-principles calculations can obtain bandgaps, linear and NLO properties, and enable theoretically guided DUV NLO material design.

**References**

1. Kang, L. *et al*. *Sci. China-Mater.* **63**, 1597-1612 (2020).
2. Kang, L. *et al*. *Acc. Chem. Res.* **53**, 209-217 (2020).
3. Payne, M. C. *et al*. *Rev. Mod. Phys.* **64**, 1045-1097 (1992).
4. Clark, S. J. *et al*. *Z. Kristallogr.* **220**, 567-570 (2005).
5. Kohn, W. *et al*. *Phys. Rev.* **140**, A1133 (1965).
6. Ceperley, D. M. *et al*. *Phys. Rev. Lett.* **45**, 566-569 (1980).
7. Perdew, J. P. *et al*. *Phys. Rev.* B **33**, 8800 (1986).
8. Perdew, J. P. *et al*. *Phys. Rev. Lett.* **77**, 3865-3868 (1996).
9. Seidl, A. *et al*. *Phys. Rev. B* **53**, 3764-3774 (1996).
10. Heyd, J. *et al*. *J. Chem. Phys.* **118**, 8207 (2003).
11. Ernzerhof, M. *et al*. *J. Chem. Phys.***110**, 5029-5036 (1999).
12. He, R. *et al*. *Appl. Phys. Lett.* **102**, 231904 (2013).
13. Kang, L. *et al*. *J. Phys. Chem. C* **117**, 25684-25692 (2013).
14. Kleinman, L. *et al*. *Phys. Rev. Lett.* **48**, 1425-1428 (1982).
15. Monkhorst, H. J. *et al*. *Phys. Rev.* B **13**, 5188-5192 (1976).
16. Kang, L. *et al*. *J. Phys.-Condens. Matter* **24**, 335503 (2012).
17. Lin, Z. *et al*. *J. Phys. D* **47**, 253001 (2014).
18. Kang, L. *et al*. *Phys. Rev. B* **102**, 205424 (2020).
19. Lin, J. *et al*. *Phys. Rev.* B **60**, 13380 (1999).
20. Lin, Z. S. *et al*. *Comput. Mater. Sci.* **60**, 99-104 (2012).
21. Godby, R. W. *et al*. *Phys. Rev. B* **37**, 10159 (1988).
22. Chen, C. T. *et al*. Nonlinear Optical Borate Crystals: Principles and Applications. (Weinheim: Wiley-VCH, 2012)
